# Supplementary material for: No association between cataract surgery and mitochondrial DNA damage with age-related macular degeneration in human donor eyes
Source: PLoS One. 2021 Oct 19;16(10):e0258803. doi: 10.1371/journal.pone.0258803 (PMC8525771; doi:10.1371/journal.pone.0258803)
Supplement: Attachment — Submitted filename: Review comments.pdf [file pone.0258803.s002.pdf]

Armbrust et al. investigated the cataract surgery status, frequency of RPE mtDNA lesions, and AMD severity. A total of 157 human donor eyes were examined. Authors hypothesized that if cataract surgery worsens AMD, human donor eyes with a history cataract surgery would have more advanced AMD or RPE mtDNA damage. As a result, only age has a significant association with MGS grade ($P<0.0001$, Table 3).
Cataract surgery are not associated with AMD severity.
They need to use donor eyes only for mtDNA lesion frequency. Other data did not necessarily require the use of human donor eyes. The main conclusion is supposed to be that there is no association between cataract surgery status and RPE mtDNA damage.

Other several concerns are below.

P5 L98; As previously described [21]…
This should be described precisely. It seems there is no new data obtained for this study. They collected mtDNA data from three previous studies; Karunadharma et al. 2010, Terluk et al. 2015, and Exp Eye Res. 2016 (21).

Methods
Limited description on mtDNA damage and lesion frequency. Not for broad readers. Please explain more about mtDNA damage experiments. Example; qPCR assay permits monitoring the mtDNA integrity directly from total cellular DNA without the need for isolating mitochondria. Lesions such as oxidative DNA damage can slow down DNA polymerase reaction. PCR will amplify normal DNA to a greater extent than damaged DNA under identical conditions. Hence, by comparing PCR amplification efficiency, DNA damage can be expressed in terms of lesions per kilobase mathematically (PMID: 11020328).

P4 L69; In support of this hypothesis…
Should be described more precisely. Previously a significant decrease in number and area of mitochondria as well as loss of cristae and matrix density were found in AMD and normal aging.

P4 L69;
Should be “mitochondrial (mt) DNA damage”?
