# Supplementary material for: No association between cataract surgery and mitochondrial DNA damage with age-related macular degeneration in human donor eyes
Source: PLoS One. 2021 Oct 19;16(10):e0258803. doi: 10.1371/journal.pone.0258803 (PMC8525771; doi:10.1371/journal.pone.0258803)
Supplement: Attachment — Submitted filename: Comments_AMD_+_cataract.pdf [file pone.0258803.s003.pdf]

“No association between cataract surgery and age-related macular degeneration in human donor eyes ”

1) The submitted manuscript investigates the prognostic significance of cataract surgery over AMD progression and mitochondrial DNA damage in the RPE, in a statistically independent manner.

The manuscript is well-presented, well-written in standard academic English, and appropriately structured. Despite some limitations related to data collection and chronological order of diagnoses, which were highlighted by the authors in the Discussion section, the proposed methodology covers all necessary parameters. The statistical data presented support the conclusions, and the combination of univariate and multivariate analysis was correctly selected and applied. Clinical significance of the statistical findings is further highlighted in the discussion, along with a concise presentation of the study limitations. Overall, the manuscript meets the criteria to be published in this journal.

2) Statistical analysis has been performed using the appropriate statistical methods and standard software. Data interpretation was performed in a similar manner, and is representative of the prognostic relevance of the conclusions reached.

3) The manuscript is presented in an intelligible fashion, and very well-written in academic English.

4) In line 158 of the results section, the authors could consider replacing “lens status” with “history of cataract surgery”. This substitution might better represent the prognostic relevance of intra-operative light exposure and ensuing inflammation that the authors are addressing with their analysis, rather than the lens status itself.
